# Dietary Patterns, Food Insecurity, and Their Relationships with Food Sources and Social Determinants in Two Small Island Developing States

**DOI:** 10.3390/nu14142891

**Published:** 2022-07-14

**Authors:** Divya Bhagtani, Eden Augustus, Emily Haynes, Viliamu Iese, Catherine R. Brown, Jioje Fesaitu, Ian Hambleton, Neela Badrie, Florian Kroll, Arlette Saint-Ville, Thelma Alafia Samuels, Nita G. Forouhi, Sara E. Benjamin-Neelon, Nigel Unwin, on behalf of the CFaH Team

**Affiliations:** 1 MRC Epidemiology Unit, School of Clinical Medicine, University of Cambridge, Cambridge CB2 0QQ, UK; divya.bhagtani@mrc-epid.cam.ac.uk (D.B.); nita.forouhi@mrc-epid.cam.ac.uk (N.G.F.); 2 The Faculty of Medical Sciences, University of the West Indies, Cave Hill Campus, P.O. Box 64, Bridgetown BB11000, Barbados; eden.augustus@gmail.com; 3 European Centre for Environment and Human Health, University of Exeter Medical School, Truro TR1 3HD, UK; E.C.Haynes@exeter.ac.uk; 4 Pacific Centre for Environment and Sustainable Development, University of the South Pacific, Suva 0101, Fiji; viliamu.iese@usp.ac.fj (V.I.); jioje.fesaitu@usp.ac.fj (J.F.); 5 George Alleyne Chronic Disease Research Centre, University of the West Indies, St. Michael BB11000, Barbados; catherine.brown@cavehill.uwi.edu (C.R.B.); ian.hambleton@cavehill.uwi.edu (I.H.); 6 Faculty of Food and Agriculture, University of the West Indies, Lewis Avenue, 686, St. Augustine 32080, Trinidad and Tobago; neela.badrie@sta.uwi.edu (N.B.); arlette.saintville@sta.uwi.edu (A.S.-V.); 7 Institute for Poverty, Land and Agrarian Studies and DSI-NRF Centre of Excellence in Food Security, University of the Western Cape, Cape Town 7535, South Africa; florian@plaas.org.za; 8 Caribbean Institute for Health Research, University of the West Indies, Mona, Kingston 7, Jamaica; alafiasam@gmail.com; 9 Department of Health, Behavior and Society, Johns Hopkins Bloomberg School of Public Health, Baltimore, MD 21205, USA; sara.neelon@jhu.edu

**Keywords:** diet, nutrition, latent class analysis, Food Insecurity Experience Scale, food consumption patterns, Pacific, Caribbean, Fiji, St. Vincent and the Grenadines

## Abstract

Small Island Developing States (SIDS) have high burdens of nutrition-related chronic diseases. This has been associated with lack of access to adequate and affordable nutritious foods and increasing reliance on imported foods. Our aim in this study was to investigate dietary patterns and food insecurity and assess their associations with socio-demographic characteristics and food sources. We recruited individuals aged 15 years and above from rural and urban areas in Fiji (*n* = 186) and St. Vincent and the Grenadines (SVG) (*n* = 147). Data collection included a 24 h diet recall, food source questionnaire and the Food Insecurity Experience Scale. We conducted latent class analysis to identify dietary patterns, and multivariable regression to investigate independent associations with dietary patterns. Three dietary patterns were identified: (1) low pulses, and milk and milk products, (2) intermediate pulses, and milk and milk products and (3) most diverse. In both SIDS, dietary pattern 3 was associated with older age, regularly sourcing food from supermarkets and borrowing, exchanging, bartering or gifting (BEB). Prevalence of food insecurity was not statistically different across dietary patterns. In both SIDS, food insecurity was higher in those regularly sourcing food from small shops, and in SVG, lower in those regularly using BEB. These results complement previous findings and provide a basis for further investigation into the determinants of dietary patterns, dietary diversity and food insecurity in these settings.

## 1. Introduction

Small Island Developing States (SIDS) comprise 58 countries and territories that share common challenges, including, but not limited to, susceptibility to natural disasters, poor infrastructure to support local food systems, low domestic food production and high reliance on imported foods, a large proportion of which are ultra-processed [1]. Of the 38 SIDS that are independent states and full members of the United Nations, 29 are located in the Pacific (*n* = 13) and Caribbean (*n* = 16) [2]. All 29 of these SIDS face a double burden of malnutrition—the co-existence of overweight and obesity, and micronutrient deficiencies [3]. In all SIDS in the Caribbean and Pacific, over one in three adults are overweight and over one in ten women of reproductive age are anaemic. In some of the poorest SIDS in the Caribbean and Pacific, which include, but are not limited to, Haiti, the Solomon Islands and Togo, more than one in five children aged under 5 years have stunting of growth [3]. In all SIDS in the Caribbean and Pacific the probability of premature (before the age of 70 years) mortality from a non-communicable disease (NCD) is high. It ranges from one in seven adults dying prematurely from an NCD (e.g., in the Bahamas) to over one in three adults (e.g., in Fiji) [4].

In SIDS, the challenges in accessing adequate, affordable and nutritious food have been linked to policies that promote the import of less healthy foods, partly driven by current World Trade Organisation (WTO) agreements that all WTO members are required to abide by. It is the Agreement on Agriculture [5], in particular, that restricts the ability of governments to support national food producers and, where necessary, protect them from competition from cheap food imports. Other factors include the challenges of economies of scale; lack of initiatives that encourage local fresh food production and consumption; and a paucity of incentives to promote agriculture and associated livelihoods, especially among the young [6]. Food imports are the largest source of food in SIDS, accounting on average for around two thirds of food consumed in those in the Caribbean and Pacific. Half of the Caribbean SIDS import over 80% of their food [7]. Imported foods generally tend to be energy dense, high in sugar, salt and fat and have poor nutritional quality [1,7]. The increasing reliance on high and ultra-processed food imports has been associated with poorer diet quality and the high and rising burden of diet-related non-communicable diseases [1,8]. Given these aforementioned features of SIDS, the UN and the Food and Agriculture Organization (FAO) consider food and nutrition security in SIDS to be an international policy priority [1].

Examining dietary patterns can provide a useful basis from which recommendations can be made to improve food and nutrition security. Dietary patterns have been investigated and studied in relation to the risk of chronic diseases; for example, diets rich in refined grains have been associated with the development of insulin resistance, whereas diets higher in fruits, vegetables, whole grains, fish and poultry have been associated with improvements in cardiovascular health and healthier body weight [9,10]. Dietary patterns depend on a range of factors including biology, culture, food availability and lifestyle [11]. Thus, it is important to not only investigate context-specific dietary patterns but to also assess them in relation to broader factors that can potentially impact dietary consumption.

Food insecurity, or the inability to obtain adequate quantity and quality of food, has been found in some studies to be associated with dietary patterns [12]. Household food insecurity in the US has been associated with high reliance on processed energy-dense foods; inadequate intake of fruits, vegetables and dairy products; lesser micro-nutrient intakes; and lower diet quality scores, especially among women [13]. A review by Larson and Story showed that in the US, the positive association between household food insecurity and overweight or obesity was largely consistent in women but inconsistent in men [14]. Another study using population representative data from Canada and the US found that food insecurity among low-income families was associated with a significantly higher prevalence of diabetes, especially among women [15,16]. Further, a study using NHANES data showed household food insecurity to be associated with markers of dyslipidemia among women but not in men [17]. Thus, evidence from the US and Canada suggests that food insecurity is associated with less healthy dietary patterns, increased risk for poor nutritional status, and adverse health outcomes, particularly in women. Little evidence exists from SIDS on dietary patterns and their relationships with risk factors for NCDs or with social determinants or food insecurity.

The work we describe here is part of the Community Food and Health (CFaH) project, where we have previously described the relationships between socio-demographic characteristics, food sources and dietary diversity in adults from two SIDS: Fiji and St. Vincent and the Grenadines (SVG). We found, for example, that dietary diversity was positively associated with regularly sourcing food through borrowing, exchanging, bartering or gifting (BEB), and negatively associated with sourcing food from a small shop [18]. In this paper we extend our analyses to investigate dietary patterns, estimate the prevalence of food insecurity and assess their associations with food sources and socio-demographic characteristics. We further assess the association between food insecurity and dietary patterns. Our work here adds to the overall goal of the CFaH project that seeks to contribute towards filling the evidence gaps on the links between food sources, diet quality and health in SIDS.

## 2. Materials and Methods

### 2.1. Study Settings and Participants

This was a cross-sectional study conducted in two independent, upper middle-income SIDS [19]—Fiji in the Pacific and SVG in the Caribbean. These countries were pragmatically chosen owing to the links research teams in the Pacific and Caribbean have with their Ministries of Health. Fiji is larger than SVG with a land area of 18,000 km^2^ and a population of 875,000. SVG has a population of nearly 109,000 and a land area of 400 km^2^ [20]. Poverty rates are high in many SIDS, including in Fiji and SVG where poverty rates are 35% and 30%, respectively. Poverty and lack of adequate employment opportunities are key constraints for access to food in SIDS [7]. SIDS have a high and increasing reliance on food imports. For example, food imports in the Pacific rose from approximately 40% to 60% between 1990 and 2011. This was similar in the Caribbean, where food imports rose from 45% to 67.5% [7].

Within Fiji and SVG, study areas were selected in consultation with local stakeholders and the Ministry of Health. We recruited households from purposively chosen urban, rural, and high and low socio-economic areas. In SVG, a convenience sample of all households within a selected area were approached, whereas in Fiji, households were numbered using satellite photographs and selected via a computer random number generator. In both Fiji and SVG, all household residents aged ≥15 years were eligible to participate.

### 2.2. Data Collection Toolkit—Development and Contextual Adaptation

A context-appropriate toolkit was administered to capture individual-level information on diet, experience of food insecurity, where and how frequently food is sourced, and socio-demographic and health data. Full details of the five-instrument toolkit, including its sources and key features, have been published elsewhere [18] and are summarised here.

#### 2.2.1. Dietary Diversity

We applied the instrument co-designed by the FAO and USAID’s Food and Nutrition Technical Assistance III (FANTA) project to generate an internationally comparable dietary diversity score (DDS), defined as the number of standard food groups consumed over a 24 h reference period [21]. As indicated by the FAO and FHI guidelines, we conducted a multiple-pass open recall to capture the range of food and drink items consumed over the previous day (from midnight to midnight). Recalled items were classified into the recommended FAO and FHI food groups [21]. Interviewers used measuring utensils and show-cards (food portion images) to aid participants in their serving size estimation and to ensure consistency in reporting. As recommended by the FAO and FHI guidelines, a standard cut-off of 5 of the 10 defined DDS food groups was used to indicate dietary diversity [21].

#### 2.2.2. Diet Screener

A diet screener was designed to complement the dietary diversity recall. Screener questions were informed by the diet component of the WHO and PAHO STEPS survey [22,23], and the regional dietary guidelines from Fiji and SVG [24,25]. Questions were aimed to capture frequency and quantity of consumption of specific food and drink items for which there is prior evidence of association with NCDs—for example, fruits and vegetables, salt, red meat, sugar-sweetened beverages (SSB) and ultra-processed foods [8,26,27,28,29].

#### 2.2.3. Food Insecurity Experience Scale

FAO’s internationally comparable and validated Food Insecurity Experience Scale (FIES) survey module was employed to measure each participant’s experience of food insecurity during the past year. FIES is a metric of severity of food insecurity that relies on an individual’s self-reported experiences and behaviours regarding their access to adequate food [30]. The FIES is the official metric used to measure success towards achievement of Sustainable Development Goal 2.1.2—prevalence of moderate or severe food insecurity in the population. The FIES consists of an eight-item questionnaire that asks participants (yes or no) if in the previous 12 months there was a time when they (1) “were worried about not having enough food to eat”, (2) “were unable to eat healthy and nutritious food”, (3) “ate only a few kinds of food”, (4) “skipped a meal”, (5) “ate less than they thought they should”, (6) “ran out of food”, (7) “were hungry but did not eat”, and (8) “went without eating for a whole day” because they did not have enough money or resources [31]. Affirmative responses to these questions were aggregated to give a raw score ranging from 0 to 8, which classifies respondents according to the intensity of food insecurity as mild, moderate and severe.

#### 2.2.4. Food Sources Questionnaire

A questionnaire was designed by the research team to capture where foods were procured and how frequently they were consumed. Participants were asked whether they produced their own food (growing, gathering, hunting or fishing), purchased their food (from different retailers), borrowed/exchanged/bartered/gifted food or received food through food aid. The questionnaire included the same food groups as those in the dietary diversity recall and diet screener [18].

#### 2.2.5. Socio-Demographic and Health Questionnaire

A series of questions from the WHO and PAHO STEPS survey instruments were used to collect information on participant age, sex, ethnicity, household size, education, employment and medical history [22,23]. Height was measured using a mobile stadiometer (Seca 2017, Hamburg, Germany) and weight was measured using a digital scale (Seca Robusta 813).

### 2.3. Data Collection

We trained ten local data collectors from Fiji and nine from SVG to administer the data collection toolkit. In Fiji, data collectors conducted interviews in iTaukei or English, as preferred by the participant. In SVG, all interviews were conducted in English, which is also the local language. Data collectors were trained by the research team who conducted training sessions on the collection of dietary and anthropometric data, aiming to standardise data collection and minimise inter-observer variation. Further, data collectors had access to training materials developed by research staff, including show-card examples of local foods for each country, presentation slides, “how to” videos and standard operating procedure manuals. Data were entered electronically in REDCap (version 7.3.4, Nashville, TN, USA), using android tablets. Dietary recall data were first recorded on paper and the data collector then classified each recalled item into a food group on REDCap.

Data were collected from August 2018 until November 2018.

### 2.4. Ethics Approval

This study was approved by the University of the South Pacific (March 2018) and the University of the West Indies (June 2018) [18]. The University of Cambridge Psychology Research Ethics Committee provided oversight by reviewing and endorsing the ethical approval given by the University of the South Pacific and the University of the West Indies. We sought permission from the Ministries of Health of Fiji and SVG for conducting the study. All participants provided written informed consent, with adolescent assent and accompanying guardian consent for respondents aged 15–18 years.

## 3. Statistical Analysis

As described, building on prior work of the CFaH project, the purpose of this exploratory study was to investigate dietary patterns and food insecurity and assess their relationships with socio-demographic characteristics and food sources. The CFaH project aimed for moderate statistical precision on nutritional adequacy based on which data were collected from 100 households each in Fiji and SVG. It was estimated that this would provide a 95% confidence interval of +/− 8% on a proportion of 50%, assuming a design effect of 1.5 [18].

### 3.1. Overview

Dietary patterns were identified in Fiji and SVG, using latent class analysis (LCA). Associations between socio-demographic characteristics, food sources, body mass index (BMI) and dietary patterns were explored using multivariable multinomial logistic regression. Here, dietary pattern was the dependent variable. As recommended by the FAO [31,32], Rasch modelling was applied separately in Fiji and SVG to estimate the prevalence of food insecurity and to classify individuals by severity of food insecurity. We assessed associations between socio-demographic characteristics, food sources, BMI and food insecurity using binomial logistic regression with food insecurity as the dependent variable. Further, we also assessed if exposure to food insecurity was associated with dietary patterns. Full details on the application and rationale of using these analytical methods are described below.

### 3.2. Dietary Patterns

A data-driven method, LCA is widely used in social sciences and more recently has been applied to investigate dietary patterns in places such as the UK, Bangladesh and Brazil [33,34,35]. We used LCA to assign individuals to the most likely latent class based on their food consumption. LCA uses maximum likelihood algorithms to identify sub-groups (latent classes) in the data that are qualitatively distinct or unobservable. In this case, latent classes were the distinct dietary patterns.

Thirteen food groups were used in the exploratory LCA model, which included the ten food groups that make up the DDS and three additional groups: savoury snacks, sweets and SSBs. LCA was performed using a stepwise approach, whereby the number of latent classes was increased by 1 class at each stage. The optimal number of classes was selected based on the lowest Akaike Information Criteria (AIC). Given the exploratory nature of these analyses, we chose the AIC as providing the most sensitive approach [36]. It was found that the best-fit model for both countries was a three-class solution based on the lowest AIC and interpretability of dietary patterns. Associations between dietary patterns, socio-demographic characteristics, BMI, food sources and food insecurity were first investigated in bivariate models, examining differences in means and proportions across the three dietary patterns. Multivariable multinomial logistical regression models were used. Variables that were associated with dietary patterns with a *p*-value <0.1 were entered into multivariable multinomial regression models, with dietary pattern as the dependent variable. A backward stepwise selection procedure was used to remove variables from the model until all variables had a *p*-value < 0.1. All multivariable models were adjusted for household clustering. Confidence intervals (95%) were calculated on all point estimates.

### 3.3. Food Insecurity

Rasch modelling was used to link FIES responses to the measure of severity of food insecurity. To enable cross-country comparability of the severity of food insecurity, the scale was equated to the global standard which is based on item parameter estimation values from over 140 countries covered by the Gallup World Poll (2014 to 2016) [31,32]. We applied the method recommended by the FAO to assign each participant a probability of experiencing moderate or severe food insecurity [31,32]. As recommended by the FAO, we performed a statistical validation of FIES data to assess if the Rasch model assumptions were met. Our findings indicate that the Rasch criteria were met for within-country analyses. However, we cannot directly compare Fiji and SVG as the equated model prevalence estimates worked for SVG only. Rasch modelling was conducted in the R software package (version R-3.6.1, Auckland, New Zealand), using the algorithm developed by the FAO [31,32]. All other analyses were undertaken using STATA (version 14, CA, USA).

Bivariate relationships between food insecurity and selected socio-demographic, anthropometric and food source characteristics were assessed. To identify statistically independent predictors of food insecurity, we performed binomial logistic regression, starting with variables that had a *p*-value < 0.1 in the bivariate analyses. A backward step process was then used to sequentially remove variables in order of the largest p value until all had *p*-values < 0.1. Models were adjusted for household clustering.

For global monitoring, FAO uses two different thresholds of food insecurity—moderate or severe food insecurity, and severe food insecurity [31,32]. In our study, the number of participants experiencing severe food insecurity was small, *n* = 41 (Fiji = 13; SVG = 28), and thus, we defined food insecurity as those experiencing moderate or severe food insecurity.

## 4. Results

Characteristics of the study populations, including dietary diversity and use of food sources in Fiji and SVG, have been previously described in detail elsewhere [18]. Briefly, we recruited 333 participants (Fiji *n* = 186; SVG *n* = 147) aged 15 years and over from 95 households in Fiji and 86 households in SVG. In both SIDS, nearly half (49%) of the participants were between 15 and 40 years of age, and over 60% were female. The prevalence of overweight or obesity, defined as a BMI ≥ 25 kg/m^2^, was high in both SIDS: 70% in Fiji and 65% in SVG.

### 4.1. Dietary Patterns in Fiji and SVG

LCA, undertaken for Fiji and SVG separately, identified three dietary patterns. Dietary pattern 1 was characterised by the lowest consumption of pulses and milk and milk products. The consumption of meat, poultry and fish; milk and milk products; and pulses increased from dietary pattern 1 to dietary pattern 3. Dietary pattern 2 was the most commonly consumed dietary pattern and was characterised by intermediate consumption of meat, poultry and fish; milk and milk products; and pulses. Dietary pattern 3 was the most diverse with a greater proportion of participants reporting consumption from various food groups. These dietary patterns are described in Table 1.

In Fiji, the least common was dietary pattern 3, in which around 12% of participants were placed. In this dietary pattern, a higher proportion of participants reported consumption of 11 of the 13 food groups than in dietary pattern 1 or 2. This included food groups such as vitamin A-rich fruits and vegetables, eggs, milk and milk products, and pulses, as well as savoury and sweet snacks. Over half of the participants were placed in dietary pattern 2, with its characteristics being largely similar to dietary pattern 1 (containing around a third of participants), but with the latter being characterised by lower proportions consuming eggs, milk and milk products, and pulses.

In SVG, 25% of participants were placed in dietary pattern 3, and they reported a more frequent intake of 9 of the 13 food groups compared to participants in dietary pattern 1 or 2. This was the case for fruits and vegetables, eggs, milk and milk products, and pulses, but not for snacks and sweets. In dietary pattern 1, in which 16% of participants were placed, lower proportions of participants, compared to dietary pattern 2, reported an intake of snacks, SSBs, milk and milk products, and pulses.

Although there were some commonalities between the dietary patterns in Fiji and SVG, as we have described, it should also be noted that there were some differences in the contents of the dietary patterns between the two settings. For example, proportions of participants reporting consumption of “other vegetables” differed quite markedly between Fiji and SVG across all dietary patterns.

In both settings, dietary pattern 3 was characterised by a higher mean DDS (Table 2). In Fiji, all participants in dietary pattern 3 had a DDS of 5 or more, and in SVG, 74.3% (95% CI 57.4, 86.1) did.

### 4.2. Characteristics by Dietary Pattern

The values (95% CIs) for socio-demographic characteristics, BMI and regular use of food sources in Fiji and SVG are shown in Table 3. Those variables that met the criterion for entry into the multivariable model are indicated.

### 4.3. Statistically Independent Predictors of Dietary Patterns

Statistically independent predictors of dietary patterns were investigated using multinominal logistic regression (Table 4), with dietary pattern as the dependent variable and dietary pattern 3 as the reference. In both Fiji and SVG there were associations between older age, rural residence, regularly sourcing food from a supermarket and by BEB when dietary pattern 1 and/or dietary pattern 2 (Table 4) were compared to the referent category (dietary pattern 3). In Fiji, there were associations with female sex and lower educational attainment and dietary patterns. In SVG, sourcing food from a formal small shop was associated with dietary patterns.

### 4.4. Food Insecurity and Dietary Patterns

The prevalence of food insecurity, defined as moderate or severe on the Food Insecurity Experience Scale, was 12.5% (95% CIs 8.3 to 17.9) in Fiji and 35.4% (27.9 to 43.5) in SVG. Exploration of statistically independent associations between food insecurity and socio-demographic, anthropometric and food source variables are shown in Table 5. In Fiji, older age and regularly sourcing food from an informal small shop were associated with higher odds of experiencing food insecurity (Table 5). In SVG, female sex and sourcing food from a formal small shop were associated with higher odds of food insecurity. In SVG, larger household size (>3 persons) and regularly sourcing food through BEB were negatively associated with food insecurity.

There was some overlap in factors independently associated with dietary patterns and food insecurity (Table 4 and Table 5), i.e., in Fiji, both were associated with age, and in SVG both were associated with regularly sourcing food from a formal small shop and BEB. However, we did not find evidence for a direct, statistically significant association between the prevalence of food insecurity and dietary patterns in either setting (Table 6), although the confidence intervals are wide.

## 5. Discussion

Understanding the relationships between dietary patterns, food insecurity, socio-demographic characteristics and food sources is important to inform the design and targeting of public health initiatives and interventions aimed at improving diet and nutrition. The primary objective of this exploratory study was to investigate in two SIDS, Fiji and SVG, the existing dietary patterns and prevalence of food insecurity and their relationships with socio-demographic characteristics and food sources. As far as we are aware, this is the first study from SIDS to use a data-driven approach (LCA) to explore dietary patterns and their relationships with socio-demographic characteristics, food sources and food insecurity.

### 5.1. Dietary Patterns and Their Associations with Socio-Demographic Characteristics, Food Sources and Food Insecurity

We identified three distinct dietary patterns in each setting. In Fiji and SVG, dietary pattern 1 was characterised by the lowest consumption of pulses, and milk and milk products, dietary pattern 2 was the most commonly consumed dietary pattern and was characterised by intermediate consumption of meat, poultry and fish, milk and milk products, and pulses, and dietary pattern 3 was the most diverse dietary pattern. Dietary pattern 3 was associated with older age, regular use of BEB and regular use of supermarkets in both settings. In Fiji, those with a primary education or less were more likely to consume dietary pattern 1, whereas in SVG, rural residents and those regularly using formal small shops were more likely to follow dietary pattern 2. We did not find any association between dietary patterns and anthropometric factors, nor did we find any association between dietary patterns and the prevalence of food insecurity.

We believe our study to be the first of its type in investigating the relationships between dietary patterns, socio-demographic characteristics, food sources and food insecurity in SIDS. Other studies from SIDS that have explored dietary patterns include a study from Trinidad and Tobago and another from the French West Indies [37,38]. The study conducted in Trinidad and Tobago in the Caribbean identified the typical dietary pattern (derived using principal component analysis (PCA)), which consisted of comparatively higher snacks, fried foods, sweets and lower starchy roots, cereals and eggs than the fruit and vegetable dietary pattern and the high-fat dietary pattern [38]. This is consistent with our findings, where a majority of those from SVG consumed dietary pattern 2 with similar consumption patterns for the food groups included. Similar to our findings from SVG in the Caribbean, another study conducted in the Caribbean island of the French West Indies found that the dietary pattern with the highest dietary diversity was associated with older age, female sex and with sourcing food from supermarkets [37].

Similar to our findings from the two SIDS, a study from New Zealand found that the healthy dietary pattern characterised by consumption of fruits, low fat milk, yoghurt, soup, tea and low intake of processed foods such as chips, pies, pastries, SSBs, white bread, etc. was positively associated with older age, female sex and a higher than primary education [39]. In contrast to our findings where we did not find any evidence for association between dietary patterns and BMI, this study found healthy dietary patterns to be negatively associated with BMI, waist circumference and food insecurity [39]. Our small sample sizes of 186 participants in Fiji and 147 in SVG as compared to 4657 participants from the study from New Zealand may have limited our ability to detect associations between dietary patterns, anthropometric factors and food insecurity. In addition, although we aimed for heterogeneity within the study samples, geographically and socio-economically, it is possible that compared to a setting such as New Zealand, diets are more similar within our study settings.

A novel important finding from our work was the independent association between dietary patterns and regularly sourcing food by BEB. The dietary pattern with higher dietary diversity (dietary pattern 3) was associated with regular use of BEB. Similarly, our previous work [18], showed regular use of BEB as a food source to be associated with a higher DDS and also discussed the importance of BEB as a means of food procurement with the potential to promote resilience to future environmental and other vulnerabilities [18]. Other studies have also identified food sharing as a significantly important culture of SIDS that must be considered for successful implementation of nutritional interventions [40,41]. Future research should expand our understanding of BEB as a source of food in SIDS and generate evidence that is needed to inform policy action to promote and sustain this culturally relevant source of food. In Lesotho, a region where food sharing is a common cultural practice, it was reported that a household gardening intervention reduced the proportion of households with low DD from 28% to 12%. The intervention also resulted in small increases in the intake of vitamin A-rich vegetables and other vegetables [42]. Thus, promotion of appropriately designed home gardening interventions could be a simple, culturally appropriate way to improve DD by home-grown foods being available for BEB.

### 5.2. Food Insecurity and Its Associations with Social Determinants and Food Sources

Our findings suggest that the prevalence of food insecurity was high in SVG, with approximately one in three adults experiencing food insecurity. Females had higher odds of experiencing food insecurity in SVG, and in Fiji, older adults were more likely to be food insecure. This has implications for policy action to decrease food insecurity, particularly among women and older adults. The prevalence of food insecurity found in SVG (35.4%; CI 27.9 to 43.5) was comparatively higher than the prevalence estimates previously reported in St. Lucia (22.2%) and the Bahamas (11%) [43,44]. However, given our small purposive sample and the variations in SIDS due to factors such as levels of local food production, food imports, GDP, national food policies, etc., caution is needed in comparing different SIDS. Furthermore, we were unable to directly compare our findings between SVG and Fiji as the Rasch modelling-equated prevalence estimates worked for SVG only.

In our previous work [18], we found that BEB was a more commonly used source of food in SVG as compared to Fiji with only 5% in Fiji reporting regular use of BEB versus 33% in SVG. We previously reported for the first time an independent association between regularly sourcing food by BEB and higher dietary diversity in Fiji and SVG [18]. In this study, we found that in SVG, the odds of experiencing food insecurity were around two and a half times lower for participants regularly using BEB as a source of food. We did not find this association in Fiji, which may have been due to a smaller proportion of participants using BEB in Fiji. We previously showed that in SVG, the regular use of BEB was strongly associated with consumption of own-food production [18]. Promotion and support of local fresh food production initiatives, particularly in SVG, may potentially contribute to reducing the high prevalence of food insecurity by provision of more own-produced food available for BEB, which in turn could potentially improve dietary diversity. A study conducted in a Greenlandic community showed that food sharing provided access to local traditional foods and was identified as an important source of food during times of stress, especially for women and older adults who rely more heavily on food sharing than other sections of the population [45]. Thus, policies directed towards supporting and sustaining food sharing networks could be invaluable for the more vulnerable, providing them with access to locally produced nutritious foods and improving food security.

In both Fiji and SVG, regularly sourcing food from small shops was associated with higher odds of experiencing food insecurity. We previously showed regular use of small shops to be associated with lower dietary diversity in Fiji and SVG [18]. However, further research is needed to understand these relationships with the use of small shops, including the choice of items, pricing and credit facilities offered by them. These findings may indicate that for the food insecure, small shops play an important provisioning role, possibly related to poverty and limited access to transport which constrains the use of supermarkets. Policies that support small shops and build links with local food production may, therefore, enhance food access in addition to securing livelihoods potentially threatened by supermarket expansion [46].

### 5.3. Strengths and Limitations

This was a relatively small cross-sectional study with a purposive sample designed to explore dietary patterns and food insecurity across rural and urban regions of Fiji and SVG. The cross-sectional study design means that we were unable to examine temporal associations and the small purposive sample limited our ability to undertake subgroup analyses. We used the FAO and FHI dietary diversity indicator as a proxy for diet quality and although this tool has advantages, a key limitation is that it has only been validated as an indicator of micronutrient adequacy in women of reproductive age [21]. Although a 24 h recall method was used to collect information on consumption patterns, only one day’s intake was recorded for each participant and hence, it is likely to miss day-to-day variation and does not necessarily reflect usual consumption.

Our study did not account for seasonality and this may have impacted our findings. Despite Fiji and SVG being tropical countries and having relatively small variations across seasons, some fruits and vegetables grow during the summer months when the temperature and humidity are higher than during other times of the year. This may affect access to and consumption of certain foods. However, we did ensure that we asked respondents to report their experience of food insecurity “during the last year”. In our study we used education as a proxy for socio-economic status; however, this may not adequately capture income and related access to food and the different sources of food procurement. Despite our attempt to ensure our sample was geographically and socio-economically diverse, it cannot be said that our sample was nationally representative.

Although most of the current literature examining dietary patterns has used PCA or factor analysis, including the very few examples we found from other SIDS, LCA is being increasingly used to investigate dietary patterns, as we described in the methods section. LCA has some strengths compared to these other approaches, including that it can be used to group individuals into mutually exclusive dietary classes using all the dietary data available. Thus, each individual is ascribed to a single dietary class, and this enables the characteristics of the classes to be examined using other person-based data, including in multivariable analyses. LCA has also been described as being less arbitrary in the choice of criteria to choose the appropriate number of classes [47]. On this last point, we acknowledge that, in line with the exploratory nature of our study, we used criteria that were sensitive rather than specific to select the classes, which may be considered both a strength and a limitation.

A key strength of our study is that we used a specifically designed, context-relevant toolkit of methods to collect information on dietary intake, food sources, food insecurity and socio-demographic characteristics in Fiji and SVG. This likely reduced the level of systematic error in data collection on behalf of the participants. Lastly, we used the FAO’s internationally comparable and validated Food Insecurity Experience Scale and Rasch modelling to calibrate each participant’s experience of food insecurity.

## 6. Conclusions

In this exploratory study, we identified three distinct dietary patterns in Fiji and SVG. Dietary pattern 1 was characterised by lower consumption of pulses, and milk and milk products, dietary pattern 2 was the most consumed dietary pattern and intermediate in consumption of pulses, and milk and milk products, and dietary pattern 3 was the most diverse. However, while emphasizing these commonalities between the dietary patterns in the two settings, it is important to acknowledge that there were differences in some of the foods consumed. The health implications of these consumption patterns or dietary patterns requires further investigation, but it can be hypothesised that the most diverse dietary pattern is likely to have greater health benefits. Dietary patterns in both settings were associated with regularly sourcing food from supermarkets and by borrowing, exchanging, bartering or gifting food (BEB) and, in SVG only, with regularly sourcing food from formal small shops. The prevalence of food insecurity was not directly associated with dietary patterns. However, in SVG, both dietary patterns and food insecurity were independently associated with regularly sourcing food from formal small shops and BEB. Our findings suggest avenues for future research. These include larger, confirmatory studies on the existence and nature of dietary patterns in these and other SIDS in the Caribbean and Pacific and their relationships to local food environments; further investigation into the underlying determinants of dietary patterns, including whether they differ by population subgroup, such as by gender; and finally, studies that aim to understand the nature of the relationships between food sources (such as small shops, supermarkets and BEB), dietary quality, food insecurity and measures of poverty. Such future research, building on the findings described here, would be invaluable in informing interventions designed to improve population nutrition in SIDS.

## Figures and Tables

**Table 1:** Results from latent class analysis based on reported consumption of 13 dietary food groups in the previous 24 h period. Figures in the table are percentages of individuals within a specific dietary pattern reporting consumption of each food group.

Food Groups	Fiji (*n* = 186)			SVG (*n* = 147)		
	Dietary Pattern 1 (*n* = 61)	Dietary Pattern 2 (*n* = 102)	Dietary Pattern 3 (*n* = 23)	Dietary Pattern 1 (*n* = 23)	Dietary Pattern 2 (*n* = 88)	Dietary Pattern 3 (*n* = 36)
Grains and roots	93.4	94.2	100	100	98.9	100
Meat, poultry and fish	68.9	74.5	94.1	87	92	94.4
Vitamin A-rich fruits and vegetables	27.3	25.5	73.7	26.1	25	37.1
Other fruits	6.8	14	45.5	8.7	11.4	13.9
Other vegetables	32.2	57.3	33.3	0	0	100
Dark-green leafy vegetables	100	0	100	17.4	6.8	27.8
Eggs	3.4	14.9	22.2	26.1	14.8	36.1
Milk and milk products	22.4	39.8	88.2	13	45.5	66.7
Nuts and seeds	0	0	100	8.7	9.1	13.9
Pulses	13.6	32.7	64.3	26.1	31.8	44.4
Savoury snacks	25.4	19	50	4.3	28.4	27.8
Sweets	15.3	28	44.4	39.1	34.1	27.8
SSB ^a^	44.8	47.6	100	0	100	100

^a^ Sugar-sweetened beverages.

**Table 2:** Dietary diversity score by dietary patterns. Figures in brackets are 95% CIs.

	Dietary Pattern 1	Dietary Pattern 2	Dietary Pattern 3
**Fiji (*n*)**	61	102	23
DDS ^a^ (Mean)	4.0 (3.6, 4.4)	4.0 (3.7, 4.3)	6.9 (5.8, 7.9)
DDS ≥ 5 (%)	31.5 (20.5, 45.0)	33.0 (24.3, 43.0)	100
**SVG (*n*)**	23	88	36
DDS (Mean)	3.6 (3.0, 4.2)	3.6 (3.3, 3.8)	5.4 (4.9, 5.9)
DDS ≥ 5 (%)	30.4 (15.2, 51.7)	20.5 (13.2, 30.2)	74.3 (57.4, 86.1)

^a^ Dietary diversity score.

**Table 3:** Examination of associations between dietary patterns, socio-demographic, anthropometric and food source variables. Figures are means or percentages (95% CIs).

	Dietary Pattern 1	Dietary Pattern 2	Dietary Pattern 3
**Fiji**			
Mean age (years) ^a^	43.4 (39.2, 47.6)	38.8 (35.4, 42.1)	47.5 (40.4, 54.6)
Female sex (%) ^a^	52.5 (40.0, 64.7)	64.7 (54.9, 73.4)	82.6 (61.6, 93.4)
Primary education or less (%) ^a^	31.1 (20.8, 43.8)	45.5 (36.1, 55.4)	52.2 (32.4, 71.3)
Household size > 3 (%) ^a^	37.6 (28.3, 47.9)	49.5 (39.4, 59.6)	12.9 (7.4, 21.4)
Rural residence (%) ^a^	36.7 (25.4, 49.6)	39.6 (30.3, 49.7)	78.3 (57, 90.7)
Mean BMI ^b^ (kg/m^2^)	29.4 (27.6, 31.2)	28.8 (27.6, 30)	30 (27.6, 32.5)
Food sources used > weekly			
Own produce (%)	68.9 (56.2, 79.2)	68.6 (58.9, 76.9)	65.2 (44.1, 81.6)
Supermarket (%) ^a^	45.9 (33.8, 58.5)	62.7 (52.9, 71.6)	78.3 (57, 90.7)
Formal small shop (%)	6.6 (2.5, 16.3)	15.7 (9.8, 24.1)	13 (4.2, 33.7)
Informal small shop (%)	19.7 (11.5, 31.6)	19.6 (13, 28.5)	17.4 (6.6, 38.4)
Food service business (%)	6.6 (2.5, 16.3)	7.8 (4, 15)	8.7 (2.2, 29.1)
BEB ^c^ (%) ^a^	4.9 (1.6, 14.3)	1 (0.1, 6.7)	26.1 (12.2, 47.4)
**SVG**			
Mean age (years) ^a^	44 (36.7, 51.2)	39.2 (35.4, 43)	44.7 (38.1, 51.2)
Female sex (%)	60.9 (40.1, 78.4)	63.1 (52.2, 72.8)	63.9 (47.1, 77.8)
Primary education or less (%)	31.8 (15.9, 53.6)	34.9 (25.5, 45.6)	27.8 (15.6, 44.5)
Household size > 3 (%) ^a^	13.3 (7.3, 23.1)	62.7 (51.2, 72.9)	24.0 (15.6, 35.0)
Rural residence (%)	56.5 (36.2, 74.9)	77.3 (67.3, 84.9)	66.7 (49.9, 80.1)
Mean BMI ^b^ (kg/m^2^)	30.3 (27.2, 33.3)	28.1 (26.7, 29.6)	27.7 (25.4, 29.9)
Food sources used > weekly			
Own produce (%)	39.1 (21.6, 59.9)	44.3 (34.2, 54.9)	50 (34.1, 65.9)
Supermarket (%) ^a^	65.2 (44.1, 81.7)	68.2 (57.7, 77.1)	83.3 (67.4, 92.4)
Formal small shop (%) ^a^	13 (4.2, 33.8)	48.9 (38.5, 59.3)	25 (13.5, 41.6)
Informal small shop (%)	21.7 (9.3, 43)	34.1 (24.9, 44.7)	30.6 (17.7, 47.4)
Food service business (%)	8.7 (2.2, 29.1)	6.8 (3.1, 14.4)	5.6 (1.4, 19.9)
BEB ^c^ (%) ^a^	4.3 (0.6, 25.5)	33 (23.9, 43.5)	50 (34.1, 65.9)

^a^ Variables entered into the first step of the multinominal regression analysis, shown in Table 4. See text for details; ^b^ Body mass index; ^c^ Borrowed, exchanged, bartered or gifted.

**Table 4:** Final results from exploratory multinominal regression, with dietary pattern as the dependent variable and food insecurity as the independent variable.

	Dietary Pattern 1 Relative Risk Ratio (95% CI)	Dietary Pattern 2 Relative Risk Ratio (95% CI)
**Fiji**		
Age (years)	**0.97 (0.95, 0.99)**	**0.97 (0.95, 0.98)**
Female sex	**0.22 (0.09, 0.59)**	**0.43 (0.33, 0.56)**
Primary education or less	**3.39 (2.24, 5.14)**	1.32 (0.61, 2.88)
Rural residence	**0.22 (0.08, 0.59)**	**0.18 (0.05, 0.68)**
Food sources used > weekly		
Supermarket	**0.47 (0.29, 0.75)**	**0.92 (0.61, 1.38)**
BEB ^a^	0.23 (0.04, 1.27)	**0.04 (0, 0.41)**
**SVG**		
Age (years)	0.99 (0.97, 1.01)	0.98 (0.97, 1.00)
Rural residence	1.32 (0.20, 8.57)	2.40 (0.87, 6.59)
Food sources used > weekly		
Supermarket	0.71 (0.43, 1.17)	**0.38 (0.25, 0.57)**
Formal Small Shop	0.40 (0.15, 1.08)	**2.37 (1.16, 4.83)**
BEB ^a^	**0.05 (0.03, 0.08)**	0.48 (0.16, 1.46)

^a^ BEB—borrowed, exchanged, bartered or gifted. Values in bold font are *p*-values < 0.05.

**Table 5:** Statistically independent socio-demographic and food source predictors of food insecurity in Fiji and SVG.

	OR	95% CI	
**Fiji**			
Age (years)	**1.05**	**1.02**	**1.07**
>Weekly informal small shop	**5.59**	**1.72**	**18.19**
**SVG**			
Female sex	3.33	**1.57**	**7.06**
House size > 3	0.33	**0.13**	**0.87**
>Weekly formal small shop	3.25	**1.33**	**7.93**
>Weekly BEB ^a^	0.39	0.15	1.01

^a^ Borrowed, exchanged, bartered or gifted; Note: Rasch criteria were met for within-country analyses. However, results from Fiji and SVG cannot be directly compared as the equated model prevalence estimates worked for SVG only. Values in bold font are *p*-values < 0.05.

**Table 6:** Food insecurity by dietary patterns in Fiji and SVG. Figures are percentages (95% CIs).

	Dietary Pattern 1	Dietary Pattern 2	Dietary Pattern 3
Fiji			
Food insecurity (%)	13.1 (6.7, 24.2)	9.8 (5.3, 17.3)	21.7 (9.3, 43.0)
SVG			
Food insecurity (%)	43.5 (25.1, 63.9)	36.4 (26.9, 47.0)	27.8 (15.6, 44.5)

Note: Rasch criteria were met for within-country analyses. However, results from Fiji and SVG should not be directly compared as the equated model prevalence estimates worked for SVG only.
